# Inside the Brain: Cerebrospinal Fluid Insights in Meningitis

**DOI:** 10.7759/cureus.67008

**Published:** 2024-08-16

**Authors:** Niharika Singh

**Affiliations:** 1 Pathology, Gandhi Medical College, Bhopal, IND; 2 Pathology, Autonomous State Medical College Kushinagar, Kushinagar, IND

**Keywords:** tuberculosis, meningeal, adenosine deaminase, cross-sectional studies, human body, lymphocytosis, spinal puncture, leukocyte count, early diagnosis, glucose

## Abstract

Background

Our study focused on meningitis, an infection that can spread through the bloodstream as a primary or secondary infection from other body parts, such as sinuses, ears, and lungs. It can affect patients who have experienced trauma or surgery, as well as those with congenital defects like spina bifida. Specifically, we examined bacterial, viral, and tuberculous meningitis (TBM) cases. The primary method for confirming the diagnosis of these types of meningitis is to analyze the cerebrospinal fluid (CSF). Early diagnosis can utilize cytological and biochemical parameters. Our objective is to determine CSF's cytological and biochemical profile in patients with these specific types of meningitis.

Methods

A study was carried out at the central pathology lab from October 24, 2017, to April 24, 2018. CSF samples from suspected meningitis patients were examined for various parameters, including hematological, biochemical, microbiological, and cytomorphological aspects and specific tests for bacterial, fungal, and TBM. The study focused on patients aged 16 and above, excluding those under 16, non-compliant patients, and individuals with specific health conditions. Data were analyzed using IBM SPSS Statistics for Windows, Version 20 (Released 2011; IBM Corp., Armonk, New York, United States), and the results were presented through the use of mean, standard deviation, and percentages. Statistical tests were utilized to compare categorical variables and mean, with a significance level of p<0.05.

Results

We included a total of 156 cases, with the mean age of presentation being 56.628 years. The male-to-female ratio was 1.0526:1. Of the patients, 81 (52.1%) had been diagnosed with TBM, had elevated adenosine deaminase (ADA) levels of 48.8733±37.43740 IU/L, and CSF lymphocytosis (99%). Additionally, cases of bacterial meningitis showed markedly raised mean total leukocyte count (TLC) of 2085.50±445.47727 cells/mm^3^ and mean CSF protein levels of 349.45±113.73105 mg/dL. The study found a significant increase in protein levels and a decrease in glucose levels in the CSF of TBM and bacterial meningitis patients compared to those with other causes of meningitis (p<0.001). Guillain-Barre syndrome (GBS) and multiple sclerosis (MS) patients had TLC and ADA within normal limits. CSF ADA level greater than 6 IU/L showed a sensitivity of 97.53% and a specificity of 96.0%, making it the most specific test. A protein level in the CSF greater than 45 mg/dL demonstrated a sensitivity of 98.78% and a specificity of 24.32%, indicating it is sensitive but less specific in diagnosing TBM. Lymphocytic predominance, defined as TLC of more than 5 cells/mm^3^ with at least 50% of the cells being lymphocytes in the CSF of TBM patients, showed a sensitivity of 97.53% and a specificity of 6.67%. CSF glucose had a sensitivity of 38.27%, making it the least reliable indicator for diagnosing meningitis.

Conclusion

The CSF analysis is the primary diagnostic method for detecting meningitis. Its cost-effectiveness is a key factor, especially for patients from lower socioeconomic backgrounds in government medical colleges in India, where access to expensive diagnostic tests is limited. The efficiency of CSF analysis for early diagnosing different types of meningitis aids in management, helping to prevent complications and fatal outcomes.

## Introduction

Cerebrospinal fluid (CSF) is a clear, metabolically active fluid between the arachnoid and pia mater. It protects the brain and spinal cord, maintains a stable chemical environment, and removes waste products of cerebral metabolism [1,2]. The composition of CSF can change in both infectious and non-infectious diseases of the central nervous system (CNS). These changes may have similar patterns in different diseases, making interpreting, diagnosing, and treating them difficult. The CSF analysis is a cornerstone in the laboratory diagnosis of meningitis. It plays a crucial role in identifying abnormalities caused by bacterial, mycobacterial, or fungal infections, thereby aiding in diagnosis and guiding initial treatment. The standard CSF evaluation is a meticulous process involving steps such as checking the opening pressure, conducting a gross examination, analyzing biochemistry, cytology, and microbiology, and performing specific immunological assays [3].

Meningitis can result in inflammation of the meninges, causing specific changes in CSF values, which occurs due to the breakdown of cerebral capillaries and the blood-brain barrier, leading to protein leakage into the CSF. Consequently, there is an increased migration of leukocytes into the CSF. Usually, the CSF contains up to 5 cells/mm^3^ of leukocytes in adults and 20 cells/mm^3^ in children. In bacterial meningitis, the total leukocyte count (TLC) is typically greater than 500 cells/mm^3^, with a predominance of neutrophils. In viral meningitis, the TLC is over 100 cells/mm^3^, with a predominance of lymphocytes [2,4]. The glucose level in CSF is usually about two-thirds of the glucose level in the blood. Chemical meningitis, inflammatory conditions, subarachnoid hemorrhage, and hypoglycemia can all cause low CSF glucose (hypoglycorrhachia). Elevated CSF glucose is not associated with any pathological process. An elevated CSF protein concentration (>45 mg/dL) is a highly sensitive indicator of CNS pathology. CSF protein levels may appear falsely elevated but do not decrease in hypoproteinemia. Characteristic CSF findings in bacterial meningitis include increased white blood cells, low CSF glucose, and elevated protein levels. The Gram stain of CSF rapidly identifies organisms, but culture is the most reliable method for diagnosing bacterial meningitis [4]. In tuberculous meningitis (TBM), the CSF typically shows elevated protein levels, low glucose levels, and increased lymphocytes [5,6]. An adenosine deaminase (ADA) level of more than 6.0 IU/L in the CSF can help differentiate tuberculous from non-TBM [6,7]. Elevated TLC distinguishes bacterial from non-bacterial meningitis. Decreased lymphocytes, abnormal glucose, and slightly elevated CSF protein levels characterize fungal meningitis. Polymerase chain reaction (PCR) assays detect enteroviruses in CSF and diagnose drug-resistant tuberculosis. Routine CSF analysis is a practical and cost-effective tool for the differential diagnosis of CNS diseases [8]. Meningitis remains a significant cause of hospital admissions in India and poses diagnostic challenges. This study aims to investigate changes in CSF's cytological and biochemical values for diagnosing meningitis.

## Materials and methods

Study design

The study was conducted in the Central Pathology Lab of Gandhi Medical College, Bhopal, India. The institutional-based prospective cross-sectional study encompassed 156 CSF samples received in the central pathology laboratory for six months from October 24, 2017, to April 24, 2018.

Data collection procedure

The patient's medical history was collected from requisition forms, departmental databases, interviews, and clinical examinations.

Sample size calculation

The sample size was meticulously calculated using the G power Version 3.1.9.6 Programme, a tool developed by Franz Faul University Kiel. This software computed the total sample size using confidence intervals, type I error, response distribution, and Z value (1.96). The formula applied was $\text{Sample size} = \frac{Z^2 \times (p) \times (1-p)}{c^2}$.

We estimated the sample size based on a 95% confidence interval, a 5% allowable error, and a meningitis prevalence of 10.23%. The initial estimated sample size was 142, and after accounting for a 10% attrition rate, we increased the sample size to 156.

Sample processing

Lumbar punctures were performed on all patients suspected of having meningitis, and a 3.0 ml CSF sample was collected in four sterile plain vials. The laboratory used the first vial for biochemistry and serology. The second vial was used for microbiology, including Gram staining, bacterial culture, and sensitivity tests. The third vial was used for hematology tests, including TLC with a normal range of 0-5 cells/cm and a differential leukocyte count (DLC). The fourth vial was utilized for cytological examination. CSF samples were processed immediately upon arrival.

Methods

Using the Neubauer counting chamber, we counted the TLC and performed the DLC on leishman-stained cytospin glass slides. We used the Biochemistry Automatic Erba Analyzer (EM-360) by Transasia, India, to determine protein, glucose, and ADA levels. Samples from the second vial were centrifuged in cases of suspicious bacterial meningitis. A sample smear was applied to a glass slide, allowed to dry, stained with Gram's method, and examined using the oil immersion lens to identify bacterial presence. Indian ink preparation of CSF was done in suspected cases of cryptococcal fungal meningitis; when testing for bacterial meningitis in CSF samples, we inoculated the samples on chocolate agar and blood agar and then placed them in a candle jar with 5-10% CO_2_ and incubated them at 37^°^C overnight. We placed CSF samples onto Sabouraud Dextrose agar to test for suspected fungal meningitis and then kept them at 37^°^C for 2-3 days to allow for incubation. We also utilized the Truelab® Duo Real Time Quantitative micro PCR Analyzer by Molbio Diagnostics, India, to perform CSF PCR testing in selected samples suspected of tubercular meningitis and viral meningitis. Protein value of more than 45 mg/dl, sugar of less than 40 mg/dl, TLC of more than 5 cells/mm^3,^ and ADA of more than six was considered pathological.

Inclusion and exclusion criteria

The study included all patients aged 16 and above in the outpatient and inpatient departments, for whom the clinician had advised them to undergo CSF examination. The study excluded patients who were under 16 years old, non-compliant, had benign or malignant brain tumors, had contraindications for lumbar punctures, such as local infections, subarachnoid hemorrhages, and raised intracranial pressure, had an had an inadequate sample, came after more than one hour, or were already receiving disease-specific treatment.

Ethical clearance

All individuals participating in this study have formally consented to participate. The Institutional Ethics Committee of Gandhi Medical College, Bhopal, India, issued approval letter no: 7620/MCI/IEC/2017.

Statistical analysis

We entered all the data into an Excel spreadsheet and analyzed it statistically using IBM SPSS Statistics for Windows, Version 20 (Released 2011; IBM Corp., Armonk, New York, United States); the results were presented as mean, standard deviation, and percentages. We utilized the chi-square test to compare categorical variables and employed ANOVA to compare the means. A p-value of less than 0.05 was considered statistically significant in all comparisons.

## Results

Out of 156 patients, there were 80 (51.2%) male and 76 (48.8.%) female patients, with a male-to-female ratio of 1.0526:1. The mean age for male patients was 57.8481±18.8310 years, and for female patients, it was 55.2500±18.82206 years (Table 1).

**Table 1:** Age and sex distribution of patients.

Sex	N	Age (range)	Mean age
Male	80 (51.2%)	17-90	57.8481±18.8310
Female	76 (48.8%)	17-87	55.2500±18.82206

**Figure 1:**
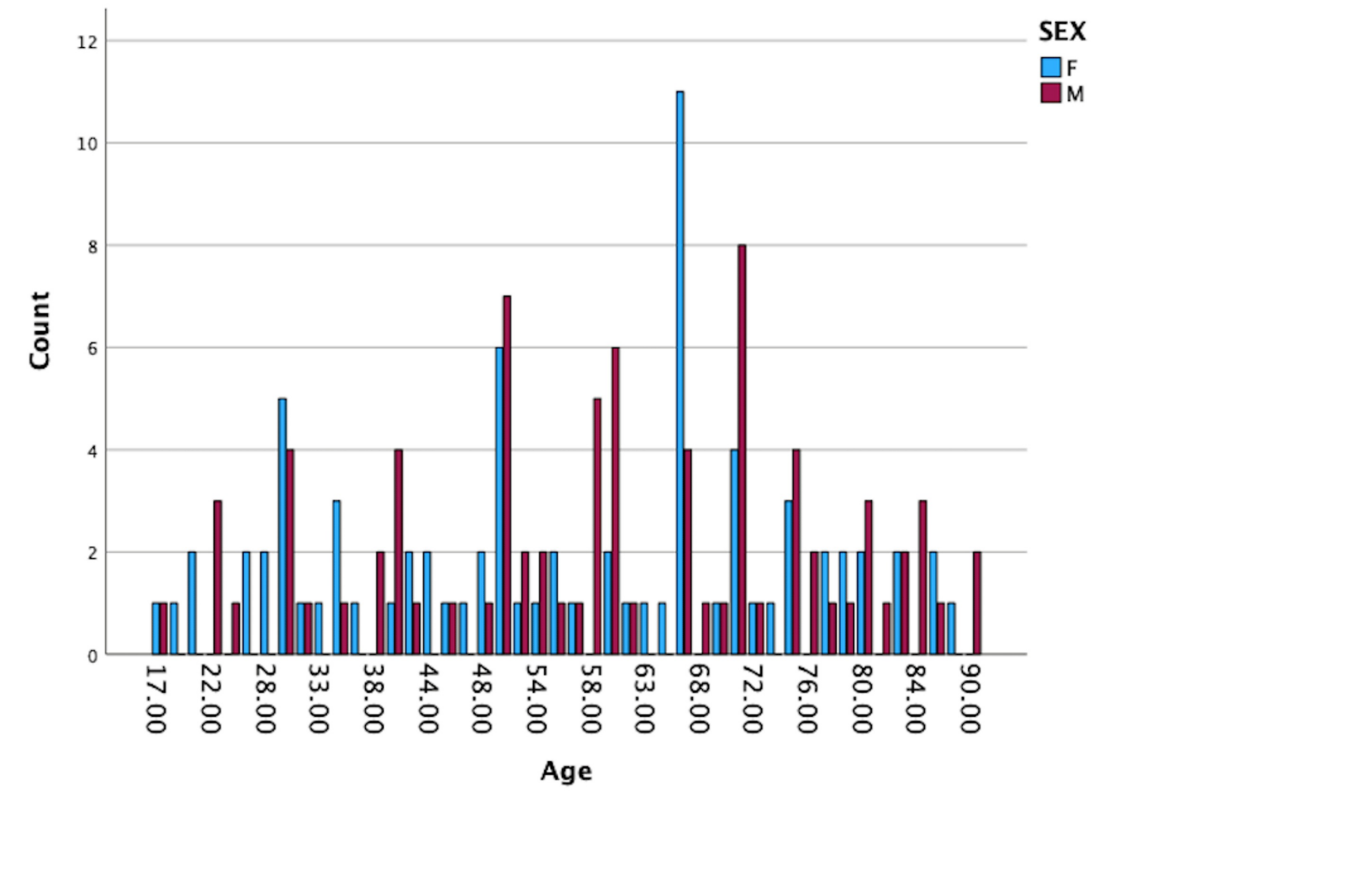
Age distribution of patients. ANOVA table; p-value=0.176

The average age of presentation was 56.628, with a minimum of 17 and a maximum age of presentation being 90 years (ANOVA table, p-value=0.176) (Figure 1).

Out of the total patients, 58 (37.10%) were diagnosed with viral meningitis, 81 (52.1%) with TBM, 2 (1.2%) with bacterial meningitis, 1 (0.6%) with fungal meningitis, and 4 (2.6%) with Guillain-Barré syndrome (GBS). Three (1.9%) patients were diagnosed with multiple sclerosis (MS). No statistical significance in the distribution of disease based on sex was seen (Pearson chi-square test, p-value=0.089) (Table 2).

**Table 2:** Distribution of diseases based on their etiology. Pearson chi-square test; p-value=0.089

Diagnosis	Male	Female	Total
Viral meningitis	23 (14.7%)	35 (22.4%)	58 (37.1%)
Tubercular meningitis	50 (32.1%)	31 (20%)	81 (52.1%)
Bacterial meningitis	1 (0.6%)	1 (0.6%)	2 (1.2%)
Fungal meningitis	1 (0.6%)	0	1 (0.6%)
Guillain-Barre syndrome	2 (1.3%)	2 (1.3%)	4 (2.6%)
Multiple sclerosis	0	3 (1.9%)	3 (1.9%)
Others	3 (1.9%)	4 (2.6%)	7 (4.5%)
Total	80 (51.2%)	76 (48.8%)	156 (100%)

The mean TLC was 185.9877±221.88401 cells/mm^3^, with a predominance of lymphocytes (99%) in all the patients with TBM. The mean TLC was significantly higher in bacterial meningitis at 2085.50±445.47727 cells/mm^3 ^(Pearson chi-square test; p-value <0.001). The diagnosis of bacterial meningitis was confirmed using Gram stain, culture, and the presence of significant pleocytosis. Viral and fungal meningitis also showed an increased TLC with lymphocytic predominance. GBS and MS patients had TLC within normal limits (Table 3).

**Table 3:** Total differential cell count in CSF in various types of meningitis and other diseases. ANOVA table; p-value<0.001 TLC: total leukocyte count; CSF: cerebrospinal fluid

Diagnosis	Mean TLC	N	Std. deviation
Viral meningitis	13.8276	58	11.40966
Tubercular meningitis	185.9877	81	221.88401
Bacterial meningitis	2085.0000	2	445.47727
Fungal meningitis	2.0000	1	0
Guillain-Barre syndrome	6.2500	4	3.94757
Multiple sclerosis	3.3333	3	1.52753
Others	7.1429	7	5.72796
Total	129.0000	156	290.23592

In TBM, the mean levels of CSF glucose were 70.8078±45.05953 mg% and 16.7±6.08112 mg% in bacterial meningitis, both significantly lower compared to the 89.7052±36.49051 mg% in viral meningitis (p<0.05). The mean protein levels in CSF were 149.9442±108.78438 mg/dL in TBM and 349.45±113.73105 mg/dL in bacterial meningitis, significantly higher compared to viral (56.2802±9.82699) and fungal meningitis (30.0300) (p<0.001). One (0.6%) patient with fungal meningitis exhibited a notable reduction in CSF glucose. In CSF, ADA levels showed a mean level of 48.8733±37.43740 IU/L in TBM. The mean levels of ADA were 3.100±2.687 and 3.0024±5.17711 IU/L in bacterial and viral meningitis, respectively (p-value<0.001) (Table 4).

**Table 4:** Biochemical profiles (glucose, protein, ADA) of various types of meningitis. ANOVA table; the p-value for CSF glucose is 0.011, and for protein and ADA is less than 0.001. ADA: adenosine deaminase

Diagnosis	Mean glucose	Mean protein	Mean ADA	N
Viral meningitis	89.7052±36.49051	56.2802±9.82699	3.0024±5.17711	58
Tubercular meningitis	70.8078±45.05953	149.9442±108.78438	48.8733±37.43740	81
Bacterial meningitis	16.7±6.08112	349.45±113.73105	3.1±2.68701	2
Fungal meningitis	1.079	30.0300	4.0	1
Guillain-Barre syndrome	66.74±24.97413	66.4925±12.71426	4.55±0.50000	4
Multiple sclerosis	61.5233±15.40923	22.2633±6.53789	3.8667±0.11547	3
Others	119.2143±123.97657	43.2286±33.24498	1.7857±1.73826	7
Total	78.5823±48.96738	107.5258±96.75266	26.8294±35.52123	156

Of all TBM patients, the Truelab® Duo Real Time Quantitative micro PCR Analyzer showed that 11 (13.58%) patients tested positive and 70 (86.42%) tested negative for CSF. We evaluated the diagnostic accuracy of CSF parameters for diagnosing TBM (Table 6). CSF ADA >6 IU/L had a sensitivity of 97.53% and a specificity of 96.0%, making it the most specific test when performed independently for TBM patients (Table 5).

**Table 5:** Diagnostic accuracy of cerebrospinal fluid adenosine deaminase in tubercular meningitis.

Statistic	Value	95% CI
Sensitivity	97.53%	91.36% to 99.70%
Specificity	96.00%	88.75% to 99.17%
Positive likelihood ratio	24.38	8.04 to 73.93
Negative likelihood ratio	0.03	0.01 to 0.10
Disease prevalence	51.92%	43.79% to 59.98%
Positive predictive value	96.34%	89.67% to 98.76%
Negative predictive value	97.30%	90.15% to 99.30%
Accuracy	96.79%	92.68% to 98.95%

A protein level >45 mg/dL had a sensitivity of 98.78% and a specificity of 24.32%, indicating that it is susceptible but less specific in diagnosing TBM (Table 6).

**Table 6:** Diagnostic accuracy of cerebrospinal fluid protein in tubercular meningitis.

Statistic	Value	95% CI
Sensitivity	98.78%	93.39% to 99.97%
Specificity	24.32%	15.10% to 35.69%
Positive likelihood ratio	1.31	1.14 to 1.49
Negative likelihood ratio	0.05	0.01 to 0.37
Disease prevalence	52.56%	44.42% to 60.60%
Positive predictive value	59.12%	55.91% to 62.26%
Negative predictive value	94.74%	71.12% to 99.25%
Accuracy	63.46%	55.39% to 71.02%

Lymphocytic predominance, defined as TLC >5 cells/mm^3^ with ≥50% lymphocytes in the CSF of TBM patients, demonstrated a sensitivity of 97.53% and a specificity of 6.67% (Table 7).

**Table 7:** Diagnostic accuracy of cerebrospinal fluid lymphocytes in tubercular meningitis.

Statistic	Value	95% CI
Sensitivity	97.53%	91.36% to 99.70%
Specificity	6.67%	2.20% to 14.88%
Positive likelihood ratio	1.04	0.97 to 1.12
Negative likelihood ratio	0.37	0.07 to 1.85
Disease prevalence	51.92%	43.79% to 59.98%
Positive predictive value	53.02%	51.28% to 54.75%
Negative predictive value	71.43%	33.33% to 92.59%
Accuracy	53.85%	45.69% to 61.85%

The CSF glucose sensitivity is 38.27%, making it a less reliable indicator for diagnosing tubercular meningitis (Table 8).

**Table 8:** Diagnostic accuracy of cerebrospinal fluid glucose in tubercular meningitis.

Statistic	Value	95% CI
Sensitivity	38.27%	27.69% to 49.74%
Specificity	93.33%	85.12% to 97.80%
Positive likelihood ratio	5.74	2.36 to 13.99
Negative likelihood ratio	0.66	0.55 to 0.79
Disease prevalence	51.92%	43.79% to 59.98%
Positive predictive value	86.11%	71.78% to 93.79%
Negative predictive value	58.33%	53.86% to 62.68%
Accuracy	64.74%	56.70% to 72.21%

## Discussion

National TB Prevalence Survey (2019-21) in India estimated a crude prevalence of TB infection at 31.3% among individuals aged 15 years and above [9]. Several studies conducted in various states of India have indicated a higher prevalence of TBM [3,10,11]. In cases of bacterial meningitis, caused primarily by gram-positive bacteria, there was a significant leukocytosis in the CSF compared to TBM. A study by Moghtaderi et al. reported a TLC of 158 cells/mm^3 ^for TBM and 1000 cells/mm^3 ^for bacterial meningitis, while Kaur et al. reported a TLC of 303.21 cells/mm^3^ for TBM [12,13]. In tuberculosis meningitis (TBM), lymphocytes were the most predominant. However, neutrophils were predominant in bacterial meningitis. The TLC was at its highest, reaching 2085 cells/mm^3^ in bacterial meningitis cases. In bacterial meningitis, the early rise of lymphocytes observed in 10% of cases can explain the elevated level of lymphocytes [14]. Thwaites et al. found that bacterial meningitis had a predominance of 90% polymorphonuclear cells, while TBM had a lymphocytic predominance (64%) [15]. In TBM and bacterial meningitis, we observed increased protein and decreased glucose levels compared to other forms of meningitis (p<0.001). These findings align with previous studies conducted in India [3,13].

We found that an ADA level >6 IU/L had 96.0% specificity, and CSF proteins had a maximum sensitivity of 97.53% in distinguishing between tuberculous and non-TBM. It also demonstrated a 96.34% positive predictive value and a 97.30% negative predictive value, consistent with the findings of studies conducted by Solari et al. and Chander and Shrestha [16,17]. These values indicate the accuracy of ADA levels in diagnosing TBM. Gupta et al. reported that a CSF ADA level of 10 U/L as a cutoff value showed 94.73% sensitivity and 90.47% specificity in differentiating TBM from non-TBM cases [7]. A protein level >48 mg/dL with a sensitivity of 96.6% indicates a diagnosis of TBM, a reliable diagnostic criterion that can be trusted. According to several studies, including ours, ADA was the best parameter in diagnosing TBM, followed by protein as the second best [4,12]. While our study provides valuable insights, some areas still require further investigation. Determining the cutoff value for the CSF ADA test is crucial for assessing its sensitivity and specificity. Although there is ongoing controversy surrounding this, it presents an opportunity for further research and consensus-building within the scientific community. Establishing a standardized cutoff for ADA values to diagnose TBM is a vital goal that requires collaborative efforts and multicenter studies involving diverse populations. Our study found a 13.58% positivity rate for nucleic acid amplification tests (NAAT) among 81 patients with TBM. A meta-analysis of NAAT showed wide variability in the performance. In-house and commercial tests have shown sensitivities below 60% [18,19]. Additionally, we detected viral meningitis in three (5.17%) patients using PCR to identify enteroviruses in the CSF, which aligns with findings from several other studies [20,21]. Indian ink preparation in CSF confirmed only one case (0.6%) of cryptococcal meningitis. PCR assays in CSF confirmed cases of cryptococcal meningitis for early diagnosis and treatment [22,23]. In our study, the mortality rate was 1.3% in patients with TBM and 0.66% in patients with bacterial meningitis. However, other studies have reported higher mortality rates, indicating the need for improved diagnostic and management strategies [24,25].

Study limitation

It's important to acknowledge the limitations of our study. We had a small sample size and used a limited quantity of CSF for routine microscopy and PCR or CBNAAT, which may have influenced the accuracy and reliability of our findings. Furthermore, ADA has no fixed standard cutoff value and can vary significantly across different laboratories, introducing potential variability in the results. Despite these limitations, our study offers valuable insights. We strongly recommend conducting a meta-analysis with a larger sample size to further confirm and build upon these findings.

## Conclusions

In conclusion, the study's findings emphasize the vital role of CSF analysis in the early and accurate diagnosis of different types of meningitis. The cost-effectiveness of CSF analysis is particularly beneficial for patients with limited access to expensive diagnostic tests, especially in government medical colleges in India. The effectiveness of CSF analysis in early diagnosis and differentiation between various types of meningitis is crucial in preventing complications and fatalities. Our findings highlight the importance of specific CSF parameters such as ADA, protein, and TLC in diagnosing meningitis.

An all-encompassing approach to CSF analysis facilitates efficient and effective management of neurodegenerative disorders, offering valuable insights and cost-effective solutions. Further research with a larger sample size and standardized cutoff values for diagnostic tests is recommended, as this will be essential in solidifying diagnostic accuracy and enabling better management strategies. This study encourages ongoing efforts to improve diagnostic and management strategies to enhance patient outcomes.
